# The Safeguards of Maternity Benefit

**Published:** 1913-08-09

**Authors:** 


### The Hospital

The Workers' Newspaper of Administrative Medicine and Institutional Life, Administration, National Insurance and Health.

No. 1415, Vol. LIV. SATURDAY, AUGUST 9, 1913. PRICE ONE PENNY

### THE SAFEGUARDS OF MATERNITY BENEFIT.

A fortnight ago we drew attention to the suggestions for improving the maternity benefit provisions of the Insurance Act which were put forward by Miss Davies and Miss Bondfield on behalf of the Women's Co-operative Guild. Their main contention that the thirty shillings, which is paid because the husband is an insured person, should be made the legal property of the wife and be payable directly to her we heartily endorsed. Since then, so rapidly do positions change where the Insurance Act is concerned, this provision has passed the Standing Committee, and will probably become law if it survives an ambuscade which is being prepared for it on Report. The effect of the latter suggested amendment is to empower the approved societies to permit either the husband or the wife to sign the receipt. If this amendment be accepted the Guild points out that the woman will not be protected at the time when she needs it most, and that her peace of mind cannot be secured so long as there is an opportunity for the husband to tamper with the money before handing it over to her. Moreover, the regimentation of the poor, into which a legislative benefit entering their home life so intimately as this may always degenerate, will be practically assured by forcing the approved society to discriminate as to the character of husband and wife, with results likely to be disastrous to their happy relations ever after. A good point is also made by the Guild in urging that, supposing that this amendment be carried, and the woman's receipt be usually taken, the alleged administrative difficulties as to the payment of the money to her are obviously surmountable. If the man's receipt be usually taken the woman's rights will not be protected any more than they are to-day.

The Guild has been encouraged to press forward this view by the addition of the two amendments to the Bill which they also favoured—namely, the striking out of the clause which allows a doctor, who is called in by a midwife to aid her in attending the wife of an insured person in an emergency to be paid his prescribed fee out of the maternity benefit, and the addition of a clause enforcing the Act's intention to accord the married woman four weeks' confinement sick-pay without having to produce medical evidence that she is unable to work during this period. On these two amendments we expressed our views very definitely in our issue of July 26 (page 511). We gave reasons indicating why the clause dealing with the doctor's prescribed fee should not be repealed, and why the arguments of the Guild on this point as expressed by the two ladies seem to us anything but weak and unconvincing. As to the four weeks' confinement benefit clause, it would, indeed, be scandalous if the Act's obvious and beneficent intention of preventing a mother's premature return to work, by promising her seven and sixpence a week if she does not do so, should be evaded by any approved society cruel or callous enough to put such technical difficulties in her way as the demanding of a medical certificate of her inability before paying the money due.

We return to this subject again not merely because the amendment of maternity benefit is at a critical stage, but also because of the coincidence which makes this tussle over a practical reform of the greatest possible importance coincide with the unprecedented flow of debate at the numerous conferences on medical subjects taking place this week. On the one hand we have the publication of the results of private work and research in many spheres; on the other an immediate practical attempt to secure the well-being of every mother in the insured class of the community. While this struggle is going on in Committee and on Report the Prime Minister is addressing the fifth annual tuberculosis conference on the campaign against consumption, and Mr. John Burns has opened the conference on infant mortality, and stated as his opinion that the discrepancy between the infant mortality of Burnley, which he gave at 171 per 1,000, and that of Battersea, which he gave at 83, was due "to the fact that in Burnley too large a percentage of mothers were at work, while in Battersea they were at home." Mr. Burns added that he held the view that "for at least four months before the child was born and for longer afterwards mothers should be mothers and not machines." One of the practical steps towards securing this result must be the safeguarding of maternity benefit. It would, indeed, be one of the ironies of fate if the very week during which London becomes the metropolis of medicine, in which the newspapers are full of medical conferences of every kind, this simple, practical safeguarding of maternity benefit were not made secure. While Ministers are talking this opportunity of doing something must not be lost.

